# Curriculum Innovation: Training the Front Line: A Neurologic Emergencies Curriculum Pilot for First Responders

**DOI:** 10.1212/NE9.0000000000200286

**Published:** 2026-01-15

**Authors:** Tamia V. Garrett, Shivika Chandra, Sushanth Aroor, Cristy Autry, Varsha Muddasani, Mahan Shahrivari, Brandy Davis, Mark Martyn, Dana M. Woodward, Amanda L. Jagolino-Cole

**Affiliations:** 1 Department of Neurology, McGovern Medical School at the University of Texas Health Science Center at Houston, TX; 2 Citizens Medical Center, Victoria, TX; and; 3 Victoria Fire Department, Victoria, TX

## Abstract

**Background and Objectives:**

Emergency medical services (EMS) clinicians play a critical role in early identification and management of neurologic emergencies yet may encounter sparse neurology-specific education. With interprofessional collaboration, we developed a multimodal, active-learning, and experiential curriculum for teaching prehospital care in a neurologically underserved setting. We evaluated curriculum feasibility and impact on prehospital neurologic assessment. The objectives of this study were to (1) recognize signs and symptoms of neurologic emergencies, aiding in the formulation of a prehospital differential diagnosis; (2) appraise patient encounter data for incorporation into prehospital medical decision making and facilitation of interprofessional communication and continuity of care; and (3) provide situation-specific prehospital care for patients with neurologic emergencies, including stabilization, medical management, triage, and disposition.

**Methods:**

We delivered a 10-month longitudinal neurologic emergencies curriculum to EMS clinicians in Victoria, TX. The program included case-based learning, simulation, team gamification, and pilot web-based bedside teaching. Participants completed preknowledge and postknowledge assessments for in-person interactive sessions and a simulation, as well as a summative knowledge assessment. Learner reactions were assessed by changes in self-perceived familiarity with neurologic emergencies. We tracked local acute stroke care metrics (monthly median door-to-needle time (DTNT), incidence of thrombolysis, and incidence of thrombectomy transfer) before and after the curriculum to assess impact on recognition and triage skills.

**Results:**

EMS clinicians with varying experience levels participated in 221 session attendances. Short-term knowledge assessment scores improved for neurovascular (60 vs 80, *p* < 0.01), neuromuscular and spinal cord (60 vs 90, *p* < 0.01), and traumatic brain injury and headache (60 vs 90, *p* < 0.01) emergencies, but not for epilepsy/syncope/dizziness/encephalopathy (both 80, *p* = 0.12), nor summatively (both 80, *p* = 0.97). Postsimulation assessment showed significant knowledge gain (40 vs 80, *p* < 0.01). No postcurriculum difference in median DTNT (49 vs 45 minutes, *p* = 0.72) nor thrombolysis administration (both 2, *p* = 0.21) was observed; transfer for thrombectomy evaluation increased (1 vs 3, *p* = 0.02). Learners reported consistently moderate familiarity with neurologic emergencies but expressed appreciation for the case-based, differential diagnosis approach.

**Discussion:**

Short-term knowledge improved in several areas of our curriculum, most notably with simulation. Increase in thrombectomy transfers suggests potential impact on recognition and triage. Challenges included attendance amid clinical responsibilities and sample size. Long-term knowledge retention strategies, including ongoing simulation, may help sustain gains.

## Introduction

Emergency medical services (EMS) clinicians are often the first medical professionals to assess patients with neurologic emergencies, yet they may receive a paucity of focused training in how to approach management in these scenarios.^1,2^ The National Highway Traffic Safety Administration of the US Department of Transportation National EMS Education Standards includes recommendations for neurologic emergency educational content, including stroke, syncope, spinal cord and brain injury, seizure, headache, and meningitis. However, in the setting of the myriad of other necessary training requirements and critical medical knowledge needed outside neurology, these sections are sparse and minimal guidance is provided on content delivery methods.^3-5^

EMS provider–targeted educational interventions can lead to increased knowledge and improved care of persons with acute neurologic illnesses. EMS personnel who reported receiving training in large vessel occlusion stroke assessment demonstrated increased knowledge about large vessel occlusion compared with those who did not.^2^ A community education initiative in East TX, including medical professionals such as paramedics, was associated with an increased proportion of thrombolysis receipt among patients with acute stroke.^6^ A training tool addressing status epilepticus in pediatric patients increased prehospital benzodiazepine utilization.^7^

However, previously described EMS educational interventions for neurologic emergencies largely address stroke care, rarely management of seizure. EMS personnel have reported a desire to receive more training in prehospital neurologic assessment, potentially incorporating peer collaboration, feedback, and case sharing.^1^ While individual EMS settings vary in structure and clinical responsibilities, there is room for guidance on best approaches for delivering neurology education to EMS clinicians, whose patient care responsibilities and workflows are unique compared with those of other medical professions. While many other medical professionals who manage patients with neurologic emergencies have mandatory clinical rotations and bedside, case-based learning during training, these are not standards for EMS clinicians before independent practice.

To understand better the educational needs of EMS clinicians, we performed a targeted needs assessment wherein we surveyed first responders on their learning preferences.^8^ We found that 2% of the 23 respondents reported having no previous training in management of neurologic emergencies. Among those who had previous training, 29% reported either in-person or virtual didactic experiences while only 18% reported learning during patient care in clinical practice. Participants preferred case-based, patient-centered, hands-on learning (50%) over auditory talks (20%), and visual aids and textbook reading (15% each). Based on their comments, respondents preferred in-person over virtual teaching.

First responders are essential in bridging the community and the emergency department. To support this important role, we formed an interprofessional collaboration among clinician-educator neurologists, EMS clinical and educational leaders, and a stroke coordinator to create a prehospital neurologic emergencies curriculum for EMS clinicians. Based on a targeted needs assessment and previously reported learner preferences in this setting,^1,8,9^ we aimed to implement an experiential curriculum in an active learning environment. We used experiential learning to allow participants to build on their unique sets of clinical experiences,^10^ addressing varying levels of experience and rank among EMS clinicians. Key components of this multimodal, active-learning environment included interactive case-based learning, simulated cases, and gamification.^10,11^ Our curriculum addressed emergencies that occur in multiple neurologic disciplines. We sought to address levels 1, 2, and 4 of the Kirkpatrick model of learning,^12^ evaluating curriculum feasibility and impact on knowledge, familiarity with neurologic diseases, and clinical outcomes in a neurologically underserved community.

### Objectives

Learner objectives included the following:

1. To recognize signs and symptoms of neurologic emergencies, aiding in formulation of a differential diagnosis in the prehospital setting
2. To appraise patient encounter data in the prehospital setting for incorporation into prehospital medical decision making and facilitation of interprofessional communication and continuity of care
3. To provide situation-specific prehospital care for patients with neurologic emergencies, including stabilization, medical management, triage, and disposition

Objectives were shared with learners and addressed neurovascular emergencies, traumatic brain injury (TBI) and headache, disorders of consciousness (including seizure, syncope, dizziness, and encephalopathy), and spinal cord and neuromuscular emergencies.

## Methods

### Learning Environment

We developed and implemented a neurologic emergencies curriculum for EMS clinicians in VIC, TX, in collaboration with local stroke center leadership, EMS training leadership, and neurologists from an academic tertiary care center in Houston, TX. Victoria is a neurologically underserved city with a population of approximately 65,000 at the time of the curriculum implementation.^13^ It is located 127 miles from Houston. There are 2 designated primary stroke centers, both of which rely on teleneurology for clinical care.^14^ At the time of curriculum implementation, Victoria Fire Department comprised approximately 100 first responders: approximately 20% with paramedic licensure and approximately 70% who operated as medics. Six stations serve Victoria County and respond to nearly 9,500 EMS service requests yearly, including areas surrounding the city of VIC.^15^

Our goals were for the curriculum to be interprofessional in collaboration, longitudinal, needs-based, and active and experiential in its educational approach. The curriculum was informed by both general and targeted needs assessments.^3-5,8^ We modified the originally planned structure of the curriculum from interspersed in-person and virtual educational sessions to entirely in-person sessions.^8^ Educational sessions needed to occur during EMS clinicians' shifts to reduce potential burnout and avoid off-duty work.

### Educational Content

**Figure 1:**
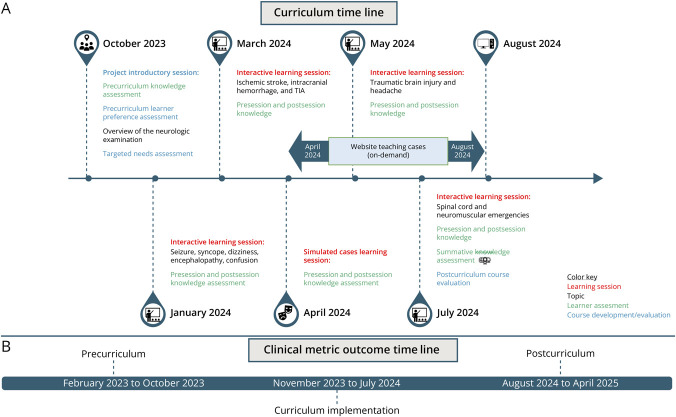
Time Line Illustrating Curriculum Implementation Session topics (A) and phases (precurriculum, curriculum implementation, and postcurriculum) used for clinical outcome analysis (B). Note: given no stroke codes between the October 2023 session and November, the clinical outcome phase started in November 2023.

We initially derived curriculum content topics from the book “Prehospital Care of Neurologic Emergencies.”^16^ Subsequently, we confirmed topics with local EMS training and clinical leadership, as well as stroke center leadership, to ensure that we included the most common scenarios EMS clinicians encounter in the field. These were as follows: dizziness, syncope, TBI, ischemic stroke, intracranial hemorrhage, TIA, seizure, spinal cord emergencies, neuromuscular emergencies (including inflammatory demyelinating polyneuropathy and neuromuscular junction disorders), headache, and encephalopathy (including toxic and metabolic types). Topics were combined for feasibility of in-person learning. Figure 1A illustrates the curriculum time line and when assessments and surveys occurred. Implementation required interdisciplinary buy-in, collaboration, and preplanning from the Victoria Fire Department, a local primary stroke center (Citizens Medical Center), and clinician educators from an academic tertiary neurologic care center.

### Curriculum Structure and Learner Assessments

Figure 1 illustrates the curriculum time line and structure, including learning sessions, learner assessments, and course evaluations. The introductory session and the 4 interactive learning sessions focused on 4 neurologic topic areas:

1. Ischemic stroke, intracranial hemorrhage, and TIA
2. TBI and headache
3. Seizure, syncope, dizziness, encephalopathy, and confusion
4. Spinal cord and neuromuscular emergencies

Before the introductory session, a baseline targeted needs assessment, precurriculum knowledge assessment, and precurriculum learner reaction and familiarity assessment were administered. During the introductory session, a neurologist guided the large group in performing the neurologic examination. The large group was then divided into smaller groups of 3–4 EMS clinicians to practice the neurologic examination on each other, with oversight from a neurologist, EMS clinical and educational leader, and a registered nurse who also serves as a stroke coordinator. Instruction and feedback provided during the small-group work on neurologic examination sought to support learner knowledge and skill development. After the introductory session, 4 interactive learning sessions were delivered longitudinally. We held each topic session 3–4 times within a week's span of the scheduled month, to include EMS clinicians covering different shifts. Interactive learning sessions comprised an icebreaker question, 2 cased-based discussions on designated neurology topics, and a team-based game reflecting content from the case-based discussion. A copy of *Prehospital Care of Neurologic Emergencies*^16^ textbook was provided to the 6 main fire stations for in-house use.

To assess session-level Kirkpatrick level 2 outcomes, we compared learner performance on a 10-question knowledge acquisition assessment provided before and immediately after each of the interactive learning sessions. Assessments addressed salient points and next clinical steps using short clinical scenarios (eTable 1).

After the last interactive learning session, we compared learner self-perceived familiarity of the neurologic topics with their familiarity before course implementation. Self-perceived familiarity of the neurologic topics was measured using a Likert scale (i.e., 1 = none at all, 2 = a little, 3 = a moderate amount, 4 = a lot, 5 = a great deal). At that time, learners completed a summative knowledge acquisition assessment addressing content spanning the entire curriculum. Self-perceived familiarity and knowledge assessments were included to address Kirkpatrick level 2. We also evaluated learner reactions to the curriculum as an overall course evaluation and to address Kirkpatrick level 1 outcomes.

Throughout the course, participation in surveys and knowledge assessments was optional and anonymized.

### Large-Group Simulation Teaching and Assessment

At the midpoint of the curriculum, we conducted a large-group learning and assessment session, which involved 3 simulated patient encounters. Simulated encounters integrated the various differential diagnoses that were taught in the course. Cases for the simulated learning session were developed by the neurologists. Program coordinators, administrators, a neurology resident, and a neurology attending served as patients during the simulated encounters. An informal large-group debrief was held after each of the simulated cases to review salient themes and provide group feedback on formulation of a differential diagnosis, administration of prehospital treatments, and retrieval of case-specific data in the field to arrive at a diagnosis, treat the patient, and facilitate interprofessional communication and continuity of care at hand-off on arrival in the emergency department. Knowledge was assessed before and immediately after the simulation session with a 10-question quiz, to assess Kirkpatrick level 2 outcomes (eTable 2).

### Webside Learning Pilot

We piloted feasibility of “webside” (web-based bedside) teaching by beaming into ambulance-designated smart phones through telehealth and assessing patients alongside EMS clinicians in the prehospital setting. Owing to logistic constraints, these were implemented late in the curriculum and involved only a small subset of learners (n = 4). In this study, we report our observations on the feasibility of implementation. For these patient encounters, EMS clinicians received clinical instruction from the clinician-educator neurologist through a HIPAA-compliant application (Teladoc^17^). EMS clinicians self-initiated these encounters when suspecting a neurologic emergency in the field.

### Clinical Outcomes

With the intention of addressing Kirkpatrick level 4 outcomes, we evaluated changes in clinical outcomes at a local primary stroke center. We compared acute stroke metrics relevant to the curricular material covered in the course as a proxy for changes in recognition of the time sensitivity and signs and symptoms for acute stroke. We focused on clinical metrics used for stroke from large vessel occlusion (i.e., monthly median door-to-needle time (DTNT), monthly incidence of thrombolytic administration, and monthly incidence of transfer for thrombectomy). Tenecteplase was used for thrombolysis. Clinical data were provided by the primary stroke center. Acute “code” strokes occurring in the inpatient setting were excluded because they would not reflect EMS provider workflow.

### Statistical Analysis

To evaluate the effects of system changes on stroke care metrics over time, we performed both nonparametric group comparisons and interrupted time-series trend analyses. The study time line was categorized into 3 phases, each lasting 9 months: precurriculum (February 2023–October 2023), curriculum implementation (November 2023–July 2024), and postcurriculum (August 2024–April 2025), to assess longer term outcomes (Figure 1B). For each clinical outcome of interest, we conducted Wilcoxon rank-sum tests to compare distributions between (1) precurriculum and postcurriculum, (2) precurriculum and implementation, and (3) precurriculum and combined implementation + postcurriculum phases. To assess temporal trends, we created a monthly time index and applied Spearman rank correlation analyses to examine the association between time and each outcome variable. These correlations were calculated separately for the precurriculum, postcurriculum, and combined implementation + postcurriculum periods to identify potential trends in each phase. A *p* value <0.05 was considered statistically significant. All analyses were conducted using R software (version R 4.5.0).

Knowledge quizzes and familiarity surveys were developed and distributed using QualtricsXM.^18^ Participants had the option of completing the knowledge assessments and surveys either on a printed paper copy or online using a Quick Response code unique to the quiz/survey. Results from all surveys and knowledge assessments were anonymized for the study team. After data were abstracted, the postsession assessment scores were applied toward continuing education credits for EMS clinicians who opted in.

### Standard Protocol Approvals, Registrations, and Participant Consents

This study was exempt on review by the UTHealth Houston Committee for the Protection of Human Subjects (HSC-MS23-0833), requiring formal permission letters from both Victoria Fire Department and Citizens Medical Center leadership, which did not have independent Institutional Review Boards.

### Data Availability

Anonymized data not published within this article will be made available by request from any qualified investigator.

## Results

### Attendance

There were a total of 221 attendances for the interactive learning sessions and the large-group simulation session. A median of 33 (range 30–41) participants attended the interactive neurology didactics. Forty-eight participants attended the in-person simulation session.

### Learner-Perceived Familiarity

**Figure 2:**
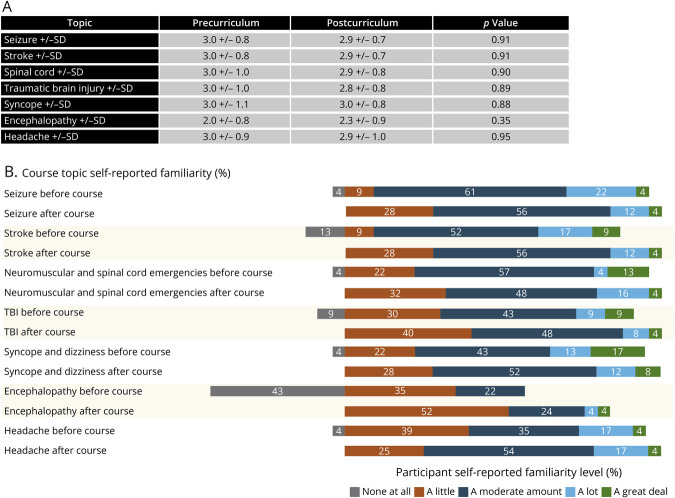
Participants' Self-Reported Familiarity With Neurology Topics (A) Familiarity in precurriculum and postcurriculum phases. (B) Demonstration of shift in reporting of familiarity as “none at all.” TBI = traumatic brain injury.

Participants reported a moderate level of familiarity with topics including seizure, stroke, spinal cord, TBI, syncope, and headache both before and after the curriculum (Figure 2A). They reported low familiarity with encephalopathy both before and after the curriculum. There were no significant differences in the level of familiarity before and after the curriculum, although the percentage of respondents reporting no familiarity decreased to 0% for all of the topics (Figure 2B).

### Learner Perceptions of the Curriculum

On the final day of the curriculum, 25 learners completed the optional course evaluation. Thirty-six percent reported less than 5 years of experience as first responders, 28% reported 5 to 10 years, and 36% reported over 10 years. The most commonly reported comments on what was useful from the course were learning about the signs and symptoms to watch out for in neurologic emergencies, as well as differential diagnoses. Participants also found the step-by-step case studies and learning about approaches to management to be useful. One participant commended that discussing rationale behind management approaches was particularly useful. Participants reported wanting to learn more about brain injury and headache topics, definitions, and medications. One participant requested online classes between the in-person classes.

### Presession and Postsession Knowledge-Based Assessments

**Figure 3:**
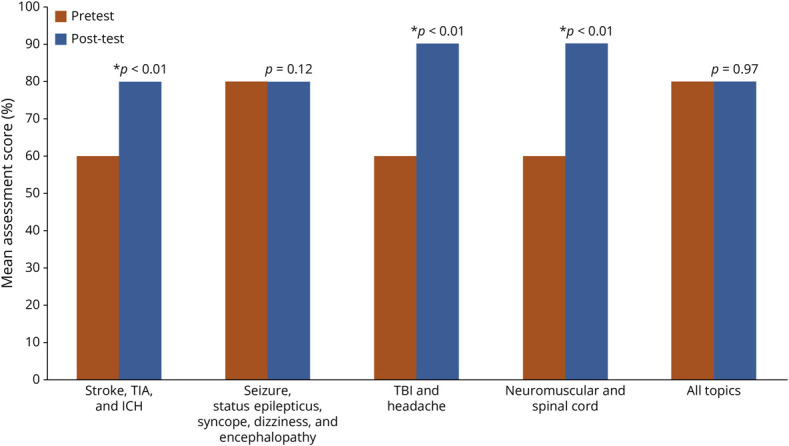
Trends in Median Pretest and Post-Test Scores by Curriculum Topic *p < 0.01, ICH = intracranial hemorrhage; TBI = traumatic brain injury.

The differences in scores between presession and postsession assessments varied depending on the topic (Figure 3). Test scores improved for the spinal cord and neuromuscular disorders session (60 vs 90 points, *p* < 0.01, 21 vs 25 respondents, respectively); TBI and headache session (60 vs 90 points, *p* < 0.01, 25 vs 29 respondents, respectively); and ischemic stroke, intracranial hemorrhage, and TIA session (60 vs 80, *p* < 0.01, 38 vs 39 respondents, respectively). There was no difference between postsession and presession assessment scores for the seizure/syncope/dizziness/encephalopathy session (80 vs 80, *p* = 0.12, 39 vs 34 respondents, respectively). The summative assessment score for the duration of the entire curriculum did not change (80 vs 80, *p* = 0.97, 31 vs 22 respondents, respectively).

### Simulation Session Knowledge-Based Assessment

Three neurologic emergency cases spanned topics including intracranial hemorrhage, ischemic stroke, seizure, encephalopathy, and headache. A presimulation and postsimulation session knowledge assessment demonstrated improved scores (40 vs 80, *p* < 0.01, for 20 respondents).

### Clinical Results

**Figure 4:**
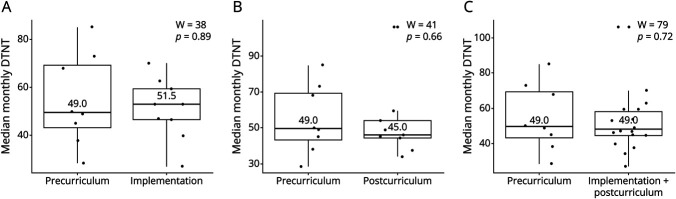
Monthly Median Door-To-Needle Time (Minutes) Surrounding the Neurologic Emergencies Curriculum Comparisons of (A) precurriculum phase with curriculum implementation, (B) precurriculum phase with postimplementation phase, and (C) precurriculum phase with combined implementation and postcurriculum phase. DTNT = door-to-needle time; W=Wilcoxon rank-sum test coefficient.

The monthly median DTNT before the curriculum did not change after its initiation (49 minutes each, W = 79, *p* = 0.72) (Figure 4). Although the postcurriculum period demonstrated a four-minute decrease compared with the precurriculum period, this was not significant (49 vs 45 minutes, W = 41, *p* = 0.66). The slight increase during curriculum implementation compared with the precurriculum period was also not significant (49 vs 51.5 minutes, W = 38), *p* = 0.89). Over time, DTNT did not change (ρ = −0.03, *p* = 0.90).

**Figure 5:**
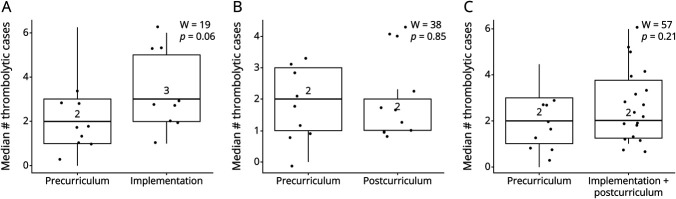
Median Monthly Incidence of Thrombolysis Surrounding the Neurologic Emergencies Curriculum Comparisons of (A) precurriculum phase with curriculum implementation, (B) precurriculum phase with postimplementation phase, and (C) precurriculum phase with combined implementation and postcurriculum phase. W=Wilcoxon rank-sum test coefficient.

Median monthly incidence of thrombolysis also did not change significantly among the different phases of the curriculum (precurriculum vs postcurriculum, 2 each, W = 38, *p* = 0.85; precurriculum vs combined implementation/postcurriculum, 2 each, W = 57, *p* = 0.21) (Figure 5). Although there was 1 additional thrombolytic case during curriculum implementation, this increase was not significant (2 vs 3, W = 19, *p* = 0.06). Incidence of thrombolysis did not change over time (ρ = −0.32, *p* = 0.20).

**Figure 6:**
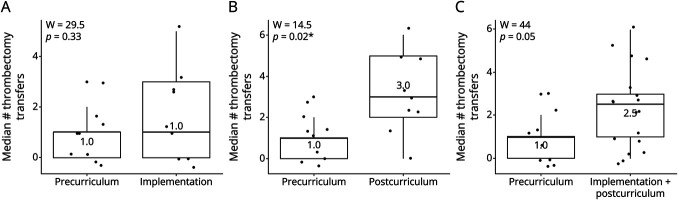
Median Monthly Incidence of Transfer for Thrombectomy Surrounding the Neurologic Emergencies Curriculum Comparisons of (A) precurriculum phase with curriculum implementation, (B) precurriculum phase with postimplementation phase, and (C) precurriculum phase with combined implementation and postcurriculum phase. *p < 0.05, W=Wilcoxon rank-sum test coefficient.

Median monthly incidence of patient transfer from the primary stroke center to a comprehensive stroke center for thrombectomy evaluation increased from precurriculum to postcurriculum (1 vs 3, W = 14.5, *p* = 0.02), Figure 6. Median incidence of transfer for thrombectomy did not change during implementation of the curriculum (1 each, W = 29.5, *p* = 0.33). There was a nonsignificant increase when combining the implementation and postcurriculum phases (1 vs 2.5, W = 44, *p* = 0.05), which seemed to be driven largely by the postcurriculum change. Incidence of transfer for thrombectomy evaluation over time increased after curriculum initiation, but not significantly so (ρ = 0.42, *p* = 0.08).

### Webside Teaching Feasibility Pilot

Webside teaching encounters were limited to 4 months of the curriculum because of setup and logistic delays. Neurologists successfully connected through telehealth to one of the fire department-issued smartphones 4 times. All encounters were for patients with acute stroke symptoms, with an average call length of 9 minutes. Feasibility limitations included short intervals from patient pickup to emergency arrival, EMS clinicians forgeting to alert the neurologist to initiate the encounter while managing patients, encounter alerts not being sent, and encounter alerts not being received on time. Connectivity and technological issues did not limit encounters.

## Discussion

Our needs-based, interprofessionally developed, multimodal, experiential, and active pilot curriculum on neurologic emergencies for EMS clinicians was feasible to implement and improved knowledge of common neurologic emergencies encountered by first responders.

We noted short-term retention of neurologic topics with knowledge-based assessment scores for stroke/TIA/Intracranial hemorrhage; neuromuscular and spinal cord emergencies; and TBI and headache, but not for epilepsy/syncope/dizziness/encephalopathy, nor for the course summatively. This is similar to knowledge retention reported in a previous educational intervention comprising a web-based training module and assessment that led to improved EMS stroke recognition, hospital prenotification, and thrombolytic treatment times, without sustained outcomes.^19^ Although this EMS-focused educational intervention addressed stroke specifically, it was similar to our curriculum in its involvement of critical and interprofessional stakeholders during its development. Inclusion of actual patient encounter feedback to EMS clinicians was an important aspect of their curriculum, although it did not yield long-term improvements in stroke recognition.^20^ The editorial accompanying this study attributes this to skill degradation, in part dependent on the initial encoding of stroke concepts in EMS training and also on a general infrequency of stroke encounters. They endorse spaced learning and multimodal teaching as mitigation strategies. Future curricula might use additional interspersed learning activities or booster simulations to reinforce key concepts in the long term. There was no difference between the presession and postsession knowledge assessment scores for the epilepsy/syncope/dizziness/encephalopathy session. The presession scores were higher than those of other interactive learning sessions for this topic, implying increased familiarity with seizure before the curriculum. EMS clinicians can initiate antiepileptic medications in the prehospital setting, but not necessarily other acute neurologic medications. However, the participants reported low familiarity with encephalopathy, which was included in the session on seizures, possibly limiting interpretation.

There were no differences in self-perceived familiarity with the core neurologic emergency topics before and after the course. Given the anonymous and optional nature of participation, it is unclear how many individual learners completed both the precurriculum and postcurriculum familiarity surveys. However, compared with the initial needs assessment,^8^ we found a similar distribution of experience with neurologic emergencies among EMS clinicians in the postcurriculum evaluation. Therefore, the groups may have been comparable. In the needs assessments, respondents reported 2–3 neurologic emergency encounters per month.^8^ Ideally, a prehospital emergencies curriculum in a neurologically underserved area would supplement field encounters to improve first responder familiarity with the subject matter. We did note that in the postcurriculum familiarity assessment, the percentage of respondents who reported having no experience at all in all topics decreased to 0 (Figure 2B).

We used a multimodal approach to the curriculum to facilitate an active learning environment. The structure of interactive learning sessions incorporated an icebreaker to set the tone for a collaborative learning environment among EMS clinicians with different levels of experience and among the educators from different medical professions. In response to our targeted needs assessment and previously reported learning preferences among EMS professionals, we incorporated case-based teaching, which may have been useful to illustrate important aspects of clinical presentations and differential diagnoses.^8,21^ Team-based games may have helped activate concepts learned from the cases and may have promoted collaboration within the small teams and some competition between groups.^22^ The multimodal nature of the curriculum seemed to appeal to learners, and case-based teaching was mentioned several times in the postcurriculum feedback. A multimodal approach to stroke education for undergraduate and graduate medical learners has been shown to increase knowledge and interest and address different levels of learning and varying learning preferences.^23^ Utilization of different teaching modalities may also reinforce core concepts.

Knowledge improved significantly after the simulation, as noted on the postsimulation assessment, reflecting short-term impact of this session. Simulation-based teaching is particularly helpful for teaching communication, workflow, situational awareness, and error prevention in the setting of complex neurologic emergencies.^11^ A simulation in the prehospital trauma setting resulted in increased motivation to learn and greater comfort in preparing for actual patient encounters for nurses.^24^ Incorporating a more formalized postsimulation debrief assessment tool in future sessions would be important to assess learners and the curriculum. A video-assisted debrief, which has been associated with improved clinical competence in a first responder trauma course, could improve feasibility of simulation performance assessment for large groups and help overcome distance barriers.^25^

Of the clinical outcomes surrounding our curriculum, the change in the number of patients transferred for thrombectomy evaluation between the precurriculum and postcurriculum phases was significant. Incorporating dedicated education on large vessel occlusion stroke workflow into the curriculum may have contributed. Historically, EMS clinicians have reported minimal exposure to training in the identification of large vessel occlusion stroke.^2^ Transport protocols for large vessel occlusion stroke vary across regions, and recommendations have evolved over the past decade.^26-28^

We found no significant difference in DTNT surrounding our curriculum; however, this metric may reflect EMS provider workflow less than other clinical outcomes. Thrombectomy exclusion data would have been helpful for interpretation. We also did not find differences in the incidence of thrombolysis administration surrounding the curriculum. It could be that, precurriculum, the EMS clinicians were more familiar with thrombolysis workflow than they were with thrombectomy workflow. In any case, evaluation of Kirkpatrick level 4 learning was limited, given many potential external factors influencing system-level clinical outcomes.

Evaluation of Kirkpatrick level 4 learning can be limited for prehospital neurologic emergencies education in a system of care wherein several factors must align for clinical management. However, it is also important to demonstrate clinical outcomes to advocate for resources and articulate value in supporting formal EMS clinician education. Our Kirkpatrick level 4 outcomes reflect only acute stroke care, and not other neurologic emergencies. Further studies may explore other acute neurologic care metrics such as timing of antiepileptic medication administration or intracranial hemorrhage care for which time sensitivity is increasingly recognized as essential,^29^ although differentiation between ischemic and hemorrhage stroke is limited without imaging capabilities. Triage metrics can be measured for all neurologic emergencies, including recognition and transportation. Information gathered in the prehospital setting needed to make critical clinical decisions in the emergency department could also be monitored to reflect curriculum effectiveness. Inclusion of emergencies in all applicable neurology disciplines is important because differential diagnoses can overlap. In the prehospital setting, with limited diagnostic capability, it can be difficult to determine the etiology of a patient's symptoms. We deployed each case-based discussion by presentation and symptomatology first, formulating differentials with the learners as new information was unveiled in the clinical scenarios.

Initiation of the pilot to assess feasibility of the webside teaching portion of our curriculum was delayed because of logistic constraints. While we experienced only a small number of webside teaching encounters, the lack of technological or connectivity obstacles for these was reassuring. Limitations occurred in coordinating initiation of the encounter and in the limited time available to teach en route to the hospital. Prehospital webside teaching can be difficult to incorporate into the already task-demanding workflow of first responders. Future curriculum iterations could focus on patient encounters originating farther from the hospital, with more travel time for teaching en route, and reaching more neurologically underserved patients.^30,31^ We had intended to longitudinally assess EMS clinician performance of the neurologic examination during prehospital webside encounters, and incorporation of formalized assessment instruments such as checklists would be important. Further studies in prehospital webside teaching can address multiple Kirkpatrick levels including reaction, learning, behavior, and results, if assessments are modeled appropriately.

We held sessions during first responder clinical shifts. Therefore, not all participants stayed for an entire session, some learners joined late, and only approximately a third of the department were able to attend sessions for any given topic because of call schedules and response to actual emergencies. It is essential for the feasibility of an EMS clinician–focused curriculum to assure that it conforms with preexisting clinical workflows and schedules. In addition, learners may have opted out of completing the presession or postsession assessments. Given the small proportion of participants, interpretation of our outcomes is limited. The small sample size also limited our ability to control survey and quiz results for level of training of the participating EMS clinicians. Anonymous attendance restricted our ability to track patterns of learner participation among the different curriculum components. We cannot exclude that any potential clinician turnover after curriculum completion may have influenced our results.

Geographic distance was certainly a barrier, with approximately 4 hours of driving roundtrip. We were fortunate to have avoided weather complications but had a backup plan to reschedule if needed. One learner did request virtual educational sessions between the in-person sessions, and a more hybrid curriculum could mitigate geographic barriers in addition to reinforcing knowledge. A previously reported EMS educational intervention incorporating small-group training sessions led to improved stroke recognition, transport time, and in-hospital mortality, although the authors note that the clinical impact of the structured education was observed in urban EMS but not in suburban settings.^32^ EMS curricular interventions should take into consideration the differences in educational needs among regions, including those in neurologically underserved and nonurban areas.

We attribute some of the success in feasibility of our curriculum to our adaptability with curricular structure and EMS clinician needs. Curriculum development and implementation must align with regional and national EMS educational requirements. Scheduling educational sessions required advanced planning and dedicated collaboration. The critical nature of clinical responsibilities among the interprofessional curriculum development team made implementation tedious at times and required a collective investment in the project. Interprofessional contributions to acute stroke care are increasingly recognized, and EMS clinicians, nurses, physicians, and other clinicians have much to learn from each other to understand clinical concepts, respective workflows, and coordination of care.^33^ We all shared the common teaching philosophy to encourage learners to understand core concepts underlying clinical decision making, rather than focusing solely on technical skills, to support the increasing role EMS clinicians have in public health and complex clinical care.^34^ A shared-leadership mindset and intentional approach to curriculum development among EMS leaders, the stroke coordinator, and the neurologists was essential.

## Glossary

DTNT: door-to-needle time
EMS: emergency medical services
TBI: traumatic brain injury
